# Knowledge about Tuberculosis: A Precursor to Effective TB Control—Findings from a Follow-Up National KAP Study on Tuberculosis among Nigerians

**DOI:** 10.1155/2017/6309092

**Published:** 2017-09-18

**Authors:** A. O. Hassan, Richard Olukolade, Q. C. Ogbuji, S. Afolabi, L. C. Okwuonye, O. C. Kusimo, J. A. Osho, K. A. Osinowo, O. A. Ladipo

**Affiliations:** ^1^Association for Reproductive and Family Health (ARFH), Abuja, Nigeria; ^2^Institute of Public Health, Obafemi Awolowo University, Ile-Ife, Nigeria

## Abstract

Tuberculosis (TB) constitutes a significant and major public health emergency globally. Nigeria is one of the 22 high burden Tuberculosis countries. A high level of community awareness and positive perception towards TB and its management is crucial for the success of any control strategy. A national baseline survey was conducted in 2008 and a follow-up study in 2012 to measure knowledge of TB among the general population. This study therefore evaluated the knowledge of the target population about Tuberculosis in the follow-up study. A cross-sectional study design was employed with a total of 3,021 respondents interviewed from six states selected randomly from each of the six geopolitical zones in the country. Quantitative and qualitative research methodologies were adopted. From the findings, about 60% of the respondents were aged between 21 and 40 years and more than half had secondary school education. Over 80% had ever heard about TB. Although there has been a significant improvement in correct knowledge of the cause of TB from baseline (19%) in 2008 to 26.5% in 2012 (*p* < 0.001), findings showed that prioritized interventions are needed to improve communication and information dissemination on Tuberculosis to the general public, to aid TB control and all prevention efforts.

## 1. Introduction

Tuberculosis (TB) constitutes a significant and major public health emergency globally. The 2015 World Global TB Report revealed that “TB still maintains the status of the world's biggest threats, due to the fact that, in 2014, the disease caused the death of 1.5 million people worldwide (1.1 million HIV-negative and 0.4 million HIV-positive)” [1]. Apart from the mortality toll from TB, the morbidity effect as indicated by the same report showed that “9.6 million people fell ill to TB in 2014 across the world (5.4 million men, 3.2 million women, and 1.0 million children), with an estimated 12% of the 9.6 million new TB cases in 2014 being HIV-positive” [1].

Nigeria was ranked as the 4th on the list of countries with the global TB burden behind India (1st), Indonesia (2nd), and China (3rd) as stated in the 2015 Global TB Report [1]. This ranking by the World Health Organization (WHO) positions Nigeria as the African country with the highest TB burden within the African continent [1]. According to the National Tuberculosis and Leprosy Control Programme statistical report in Nigeria, “90,584 of all forms of TB cases were detected in 2015” [2]. Going by the first national TB prevalence survey report (2012), “Nigeria had a prevalence rate of 323 per 100,000 for all forms of TB and an estimated incidence rate of 338 per 100,000” [3].

The national TB Control Programme covers all the thirty-six states and FCT, including all the 774 local government areas (LGAs) in the country. In terms of geographical spread, the country at the end of 2015 had “5,863 Directly Observed Treatment Short Course (DOTS) centers in the 774 LGAs” [4]. Through the DOTS expansion strategy, dissemination of TB information and services has been promoted through the training of health facilities' care providers (specifically DOTS and laboratory staff) and also through TB patients, during diagnosis and treatment initiation. However, the general population has not been well informed about TB due to the sparse use of the mass media, resulting in low awareness and sensitization about the disease among the general population.

Between 2008, when the first TB knowledge, attitude, and practice (KAP) survey was conducted in the country, and 2012, when a similar follow-up study was done, the knowledge about TB was expected to have significantly increased, considering the application of several proven interventions deployed for the implementation of TB services in Nigeria. This included the dissemination of TB messages on the mass media, printing and distribution of information, education, and communication (IEC) materials, and community sensitization during World TB Day celebrations. Others are related to the expansion of DOTS facilities and training of health workers and community volunteers, to generate demand for TB services within the communities.

The roles of the mass media in demand creation for public health services have been found to be germane to the creation of awareness and sensitization as well as in reducing stigma and discrimination. As discussed by Fakolade et al., “public health programs that focus on the use of effective mass media messages and the use of testimonials will continue to reduce stigma and discrimination” [5].

With a population of about 177,000,000 in 2014, Nigeria remains the most populous country in Africa with approximately two-thirds of the populations living in rural areas and a population median age of 17 years [6]. The country is organized as a federation, with a federal government, 36 states, and the Federal Capital Territory and 774 local government areas. The states are grouped into six geopolitical zones: northwest, northeast, north central, southeast, south-south, and southwest zones. The zones differ from each other in size, population, ecological characteristics, language, culture, settlement patterns, economic opportunities, and historical background. Wide sociocultural, economic, and geographical diversities exist across the country. There are an estimated 374 ethnic groups and more than 500 languages existing in the country. However, the predominant languages are Hausa/Fulani in the north, Yoruba in the southwest, and Ibo in the southeast. Christianity and Islam are the main religions.

## 2. Materials and Methods

This study involved a cross-sectional approach with a mixed method of both quantitative and qualitative data collection instruments. The quantitative aspect involved a household survey.

Youths, adult women, and men of reproductive age group in the communities where TB interventions have been implemented constituted the study population for the quantitative component. The sample size for the household survey was calculated using the Windows Program for Epidemiologists (WINPEPI) [7]. The applied formula was based on simple proportion with a cluster sampling technique with three key indicators: national prevalence of TB of 537/100,000 population, the mean knowledge score of TB from the baseline estimated at 62.4%, and the proportion in the baseline with correct knowledge of TB estimated at 19%. A confidence level of 95%, a precision of 0.05, and a design effect of 2 were introduced. Based on these three indicators and parameters, the minimum adequate sample size required was 472 (based on correct knowledge).

With a 10% nonresponse rate, a total of 520 respondents were estimated for each of the six states. In all, a total of 3,120 respondents were targeted in the six selected states.

A multistage sampling technique was employed. At the first stage, a state was selected from each of the six geopolitical zones in the country. The six states were selected randomly from the geopolitical zones and thereafter two (one urban and one rural) local government areas (LGAs) were selected in each state. The LGA in which the state secretariat was situated was chosen as the urban LGA while the LGA with the least population was chosen as the rural LGA. At the third stage, a list of enumeration areas (EAs) was obtained from the NPC and 10 enumeration areas (EAs) were selected randomly from each LGA making up 20 EAs per state.

At the fourth stage, a systematic sampling technique was employed to recruit twenty-six (26) participants in each EA. The procedure for selection was as follows: (a) starting from any point, the fieldworker walked around the selected EA to identify and list all households. In listing the households, the address and the name of head of households were recorded for proper tagging. All institutional households were excluded. (b) The number of eligible households in each EA was divided by twenty-six (26) to obtain the sampling interval *k*. (c) A number *j* between 1 and *k* was randomly selected using simple random sampling. The household corresponding to this number in the list of households was the first household to be included in the sample. (d) To determine subsequent households to be included in the sample, *k* was added each time. For example, *j* + *k*, *j* + 2*k*, *j* + 3*k*, and so forth, and *k* will vary from one EA to another depending on the number of eligible households.

Each of the selected households was visited and all the eligible men and women (i.e., women in the age group of 15–49 years and men aged 15–64 years) were listed by age. The person to be interviewed was selected using a table of random numbers. This means that there was no need to systematically alternate between interviewing a male and interviewing a female. Randomization ensured that we obtained a correct sex ratio for each state. A total of 3,021 questionnaires were properly completed and returned, representing a 97% response rate.

Generally, the set of questionnaires for collecting the quantitative data was designed to elicit information on respondents' knowledge, attitudes, beliefs, experiences, and practices concerning TB disease, prevention, and control in Nigeria. But more specifically, the research sought to collect information about respondents' knowledge, attitude, and practice (KAP) on the following: knowledge of TB and source of information, perception of TB prevalence in the community, routes of TB transmission, ways of preventing TB transmission, method of detection of TB, methods of treatment of TB, and attitudes towards and practices relating to TB.

The fieldworkers underwent three days of training with the data collection instruments for standardization of the interview protocol. CSPro and EpiData were used for data entry and editing. Analysis was carried out using the SPSS software.

Ethical clearance for this study was obtained from the Federal Ministry of Health's National Health Research Ethics Committee (NHREC) before the commencement of the study. Written informed consent was also obtained from all participants after the study has been properly explained to them. In this regard, the (translated) consent form was read to participants who do not understand English in their local language by trained data collection staff. Participants who were unable to write or sign after consenting to participate in the study were requested to thumb-print on the consent form. Also, verbal consent was obtained from community leaders in every community where the survey was conducted. Confidentiality was assured by ensuring that there were no personal identifiers on any data instrument, and only research personnel had access to the data. It is important to state that the data was not weighted during analysis.

## 3. Results and Discussion

### 3.1. Sociodemographic Characteristics of the General Population

Table 1 displays the sociodemographic characteristics of the survey respondents. Out of a total of 3,021 respondents from the six states where the study was conducted, 513 respondents were interviewed in Ondo, 518 in Benue, 525 in Akwa Ibom, 518 in Katsina, 424 in Ebonyi, and 523 in Gombe. The general background information of the respondents showed that there was a slightly higher proportion of females interviewed (53% females) than males. Over 60% were between the age categories of 21–40 years, while more than half (56%) had at least secondary level of education, but only 45.6% can read and write in English without difficulty. Petty trading was the commonest vocation (28%) among the respondents, followed by farming (17%) and career civil servants (14%). About three-quarters of the respondents were married (73%), while a majority were from Hausa, Igbo, and Yoruba ethnic tribes. Three out of every five persons interviewed were Christians (60%).

### 3.2. Knowledge of TB

The survey sought to understand the prevailing knowledge level of respondents on TB. This is a communicable disease caused by* Mycobacterium tuberculosis*. As an airborne disease, it is transmitted when individuals with infectious Tuberculosis cough, sneeze, talk, or spit and, by so doing, expel TB bacilli into the air; thus, transmission is rampant in crowded and poorly ventilated habitats [8].

The knowledge of TB was based on respondents' ability to recognize the cause, routes of transmission, and the methods of prevention. From the bivariate analysis, results were categorized into high and low knowledge. As shown in Table 2, a higher number of males (64.2%) than females (57.8%) had a higher level of TB knowledge, while, on the other hand, more females (42.2%) than males (35.8%) had low knowledge of TB (*p* < 0.001). This finding corroborates the results of the most recent National HIV/AIDS and Reproductive Health Survey (NARHS) in Nigeria where a higher proportion of males than females were aware of TB [9] and as discussed in the Nigerian National Demographic Health Survey [10].

Similarly, the knowledge of TB was higher among the singles (73.4%), followed by those who were married (57.5%), compared to other marital categories. This result was statistically significant (*p* < 0.001). Moreover, among all age groups, young persons aged 16 to 29 years (63.7%) had higher knowledge of TB than other age groups. This was equally statistically significant (*p* < 0.001). Respondents with tertiary education had the highest proportion (81%) of TB knowledge. This implies that young persons between the ages of 16 to 29 years had access to information on TB compared with the older categories. In addition, more persons in this age group mostly belong to the tertiary education category where high TB knowledge was observed (*p* < 0.001).

Of all the five study sites where the follow-up study was conducted, TB knowledge ranked the highest in Akwa Ibom and Ondo states at 73%. Conversely, TB knowledge was the lowest in Ebonyi state (46%). This suggests that investment in dissemination of TB information should be focused on Ebonyi, Gombe, Katsina, and Benue states. Interestingly, respondents in the formal sector had higher knowledge of TB (79%) than other occupational groups (*p* < 0.001), meaning that information about TB has not permeated the general population.

### 3.3. Attitude towards Tuberculosis: A Reflection of the Knowledge Level about TB

People's attitude towards a given disease usually reflects their level of understanding of the disease, especially at it relates to the effort made in seeking treatment. Respondents were asked to state their opinion about the likely places where treatment can be sought for TB. The self-reported responses were computed into two major responses: good and poor health seeking behavior. Those considered to have the right opinion (good) are those who indicated their willingness to seek treatment in private or government owned healthcare facilities. The negative opinion (poor), interpreted as a wrong attitude towards TB treatment in this paper, was expressed by those who considered self-medication as a panacea or prefer to seek treatment from traditional healers or spiritual or religious centers.

Table 3 provides a reflection of the surveyed population on their stance to TB treatment. The result shows that positive opinion (good) to TB treatment was expressed among respondents who had high knowledge of TB (80%) (*p* < 0.0001). Consequently, among those who had tertiary education, a high proportion (83%) indicated the right opinion about TB treatment (*p* < 0.0001) as well as among those in formal employment (81%) (*p* < 0.002).

These findings suggest that the knowledge of TB will logically inform the belief that the disease is curable which, again, can influence a positive health seeking behavior towards the disease among persons with symptoms of TB. Thus, poor health seeking behavior invokes dire consequences which in turn may fuel drug resistant (DR) TB and further complicate prevention and control efforts of TB in the country. As noted in the Global TB Report 2016, “Nigeria is one of the six countries (India, Indonesia, China, Nigeria, Pakistan, and South Africa) where progress in TB control globally depends on major advancement in TB prevention and care” [11].

### 3.4. Estimates of Crude Odds and Adjusted Odds Ratios for Overall Knowledge of TB

In an attempt to simultaneously evaluate the knowledge of TB, multiple logistic regression was employed. Knowledge of TB was the outcome variable. Firstly, Model 1 explores the crude odds, while the second model considers the adjusted odds ratios. In both models, education and employment status were associated with knowledge of TB.

Regarding Model 1, respondents with secondary education were 1.4 times more likely to have knowledge about TB than those with no formal education (OR = 1.42, *p* < 0.0001). A similar trend was observed among those in formal employment. They are 1.5 times more likely to have knowledge of TB than those not employed (OR = 1.49, *p* < 0.001).

Model 2 depicts the result after controlling for covariates (Table 4). Here, demographic characteristics and knowledge indicators were simultaneously adjusted for. The findings were similar to Model 1. Respondents with primary education were 1.4 times more likely to have knowledge about TB than those with no formal education (OR = 1.40, *p* < 0.01). Those with secondary education were 2.2 times more likely to have knowledge about TB than those with no formal education (OR = 2.16, *p* < 0.0001). Respondents with tertiary education were 2.8 times more likely to have knowledge about TB than those with no formal education (OR = 2.83, *p* < 0.0001). This suggests that persons with no formal education and formal employment have poor knowledge of TB.

## 4. Conclusions

As shown by the findings from this study, while 80% of the respondents interviewed had ever heard about TB at one point or another in their lifetime prior to the survey, a majority did not know anything about the symptoms and the causes of the disease. Only 26.5% knew the main cause of TB. This implies that the general population are ignorant about this disease. This assertion was confirmed in a similar study conducted in Edo state, south-south region of Nigeria, by Tobin et al. [12].

Moreover, comparing the baseline values about TB knowledge in the country in 2008 and the follow-up study in 2012, correct knowledge of the cause of TB has slowly increased (19.5% to 26.5%). Though statistically significant (*p* < 0.0001), this finding points to the fact that the dissemination of TB messages to the general population has not been given appropriate attention in the country due to inadequate funding on sensitization and awareness about the disease.

The positive association between education and knowledge of TB in this study was also discussed in a similar study conducted in Tanzania [13] and elsewhere in Nigeria [14]. This also suggests that knowledge about TB can be appropriately inculcated into the school curriculum to address misconceptions as proposed in a similar study conducted in Ghana [15] and also in Nigeria among secondary school students [16].

Respondents with tertiary education had the highest proportion (81%) of TB knowledge. This implies that those without formal education had no knowledge of TB. The multivariate result confirms this reality. As already shown, poor attitude towards TB treatment is a translation of poor knowledge about TB among the general population. Those with high knowledge of TB will demonstrate good health seeking behavior about TB disease (80%) (*p* < 0.0001).

Although TB treatment is free in Nigeria through donor funding support, knowledge of the disease is what will aid the uptake of TB services. When people know the cause and the symptoms of the disease, they are best informed to visit a TB treatment center when the need arises. More importantly, knowledge will mitigate social stigma generally associated with TB and other determinants of patients' delay in seeking treatment as documented in a study conducted in a chest hospital in Ibadan, southwest region of Nigeria [17], and in a related study in the southeast zone of the country, where ignorance was a barrier to the uptake of TB health services by the people [18].

On the whole, the conclusion from this study highlighted the need to develop targeted interventions to improve communication and information dissemination on the causes of Tuberculosis to the general public. Owing to the multiethnic nature of the country and considering the sharp cultural and religious divide, TB messages must be developed in a way that is culturally sensitive and acceptable with impressive clarity of messages. The messages must also be made to address misconceptions and myths about TB and must be saturated widely, especially in Ebonyi, Gombe, Katsina, and Benue states where studies indicate low awareness of TB.

The control of TB by way of detecting the cases and placing infected persons on treatment becomes easier when the target population (the general public) becomes enlightened about the disease. There is a relationship between patient's delay in seeking treatment and knowledge about the causes, transmission, and symptoms of TB as discussed by Babatunde et al. [19]. This fact has already been stressed in this paper.

In addition, community mobilization and demand creation through public education on TB are a key factor [20] that has a pertinent role to play to achieve this goal. Persons living with AIDs (PLWA) have a right to know about TB and this will reduce mortality induced through comorbid conditions [21]. On the whole, this paper recommends that public health programs through government and donor funding should make information dissemination on TB a priority to every category of Nigerians, for effective TB control and prevention and to complement ongoing interventions.

## Figures and Tables

**Table 1:** Study sites and background characteristics of respondents.

Variable	Ondo *N* = 513	Benue *N* = 518	Akwa Ibom *N* = 525	Katsina *N* = 518	Ebonyi *N* = 424	Gombe *N* = 523	Total *N* = 3021
Sex							
Males	200 (39.0)	225 (43.5)	292 (55.6)	249 (48.1)	159 (37.5)	288 (55.1)	1413 (46.8)
Females	313 (61.0)	293 (56.5)	233 (44.4)	269 (51.9)	265 (62.5)	235 (44.9)	1608 (53.2)
Age							
≤20	65 (12.7)	57 (11.0)	60 (11.4)	44 (8.5)	20 (4.7)	91 (17.4)	337 (11.2)
21–30	223 (43.5)	181 (34.9)	216 (41.1)	141 (27.2)	117 (27.6)	189 (36.1)	1067 (35.3)
31–40	139 (27.1)	132 (25.4)	120 (22.9)	155 (29.9)	126 (29.7)	123 (23.5)	795 (26.3)
41–50	61 (11.9)	77 (14.8)	91 (17.3)	115 (22.2)	117 (27.6)	66 (12.6)	527 (17.4)
>50	25 (4.9)	72 (13.9)	38 (7.2)	63 (12.2)	44 (10.4)	54 (10.3)	296 (9.8)
Education							
None	19 (3.7)	76 (14.6)	28 (5.3)	41 (7.9)	106 (25.0)	14 (2.7)	284 (9.4)
Quranic	7 (1.4)	37 (17.1)	0 (0.0)	203 (39.2)	2 (0.5)	188 (35.9)	437 (14.5)
Primary	68 (13.3)	137 (26.4)	122 (23.2)	73 (14.1)	136 (32.1)	62 (11.9)	598 (19.8)
Secondary	287 (55.9)	171 (32.9)	237 (42.1)	115 (22.2)	137 (32.3)	161 (30.8)	1108 (36.7)
Tertiary	126 (24.6)	97 (18.7)	134 (25.5)	85 (18.4)	42 (90.9)	90 (17.2)	574 (19.0)
Ability to read and write in English							
Without any difficulty	295 (57.5)	264 (50.9)	308 (58.7)	152 (29.3)	148 (34.9)	212 (40.5)	1379 (45.6)
With difficulty	171 (33.3)	134 (25.8)	160 (30.5)	105 (20.3)	138 (32.5)	145 (27.7)	853 (28.2)
Cannot	44 (8.6)	119 (22.9)	56 (10.7)	257 (49.6)	135 (31.8)	163 (31.2)	774 (25.6)
Occupation							
Schooling	97 (18.9)	32 (6,2)	88 (16.8 )	38 (7.3)	53 (12.5)	77 (14.7)	385 (12.7)
Unemployed	27 (5.3)	117 (22.5)	25 (4.8)	79 (15.3)	9 (2.1)	95 (18.2)	352 (11.6)
Petty trading	158 (30.8)	104 (20.0)	211 (40.2)	208 (40.1)	37 (8.7)	94 (18.0)	865 (28.6)
Farming	17 (3.3)	96 (18.5)	60 (11.4)	62 (12.0)	190 (44.8)	95 (18.2)	520 (17.2)
Artisan	110 (21.4)	54 (10.4)	63 (12.0)	40 (7.8)	34 (8.0)	37 (7.1)	349 (11.6)
Civil servant	71 (13.8)	107 (20.6)	58 (11.1)	80 (15.4)	36 (8.5)	69 (13.2)	411 (14.0)
Mixed	22 (4.3)	9 (1.7)	20 (3.8)	9 (1.7)	23 (2.8)	56 (10.7)	128 (4.2)
Marital status							
Single	157 (30.6)	92 (17.7)	167 (31.8)	65 (12.5)	84 (19.8)	101 (19.3)	666 (22.0)
Married	345 (67.3)	377 (72.6)	337 (64.2)	415 (80.1)	332 (78.3)	408 (78.0)	2214 (73.3)
Widowed	7 (1.4)	30 (5.8)	11 (2.1)	28 (5.4)	5 (1.2)	7 (1.3)	88 (2.9)
Divorced	1 (0.2)	8 (1.5)	4 (0.8)	8 (1.5)	1 (0.2)	2 (0.4)	24 (0.8)
Separated	2 (0.4)	10 (1.9)	2 (0.4)	1 (0.2)	1 (0.2)	0 (0.0)	16 (0.5)
Other	0 (0.0)	2 (0.4)	3 (0.6)	0 (0.0)	0 (0.0)	0 (0.0)	5 (0.2)
Missing	1 (0.2)	0 (0.0)	1 (0.2)	1 (0.2)	1 (0.2)	5 (1.0)	9 (0.3)
Ethnicity							
Hausa	2 (0.4)	72 (13.9)	3 (0.6)	483 (98.2)	1 (0.2)	144 (27.5)	705 (23.3)
Fulani	1 (0.2)	16 (3.1)	0 (0.0)	1 (0.2)	0 (0.0)	156 (29.8)	207 (6.8)
Igbo	41 (8.0)	38 (7.3)	12 (2.3)	41 (8.0)	419 (48.8)	4 (0.8)	514 (17.0)
Yoruba	438 (85.4)	13 (2.5)	0 (0.0)	438 (85.4)	1 (0.2)	8 (1.5)	460 (15.2)
Tiv	0 (0.0)	33 (6.4)	0 (0.0)	0 (0.0)	0 (0.0)	1 (0.2)	34 (1.1)
Idoma	6 (1.2)	275 (53.0)	5 (1.0)	6 (1.2)	0 (0.0)	2 (0.4)	288 (9.5)
Ibibio	1 (0.2)	1 (0.2)	348 (66.3)	1 (0.2)	0 (0.0)	0 (0.0)	350 (11.6)
Other	24 (4.7)	71 (13.7)	157 (29.9)	24 (4.7)	1 (0.2)	207 (39.4)	461 (15.3)
Missing	0 (0.0)	0 (0.0)	0 (0.0)	0 (0.0)	2 (0.5)	1 (0.2)	3 (0.1)
Religion							
Christianity	474 (92.6)	365 (70.3)	520 (99.0)	3 (0.6)	408 (96.2)	44 (8.4)	1815 (60.1)
Islam	28 (5.5)	151 (29.1)	1 (0.2)	510 (98.5)	3 (0.7)	477 (91.2)	1170 (38.7)
Traditional	8 (1.6)	3 (0.6)	0 (0.0)	3 (0.6)	12 (2.8)	2 (0.4)	26 (0.9)
Other	2 (0.4)	0 (0.0)	4 (0.8)	0 (0.0)	1 (0.2)	0 (0.0)	7 (0.2)
Missing	0 (0.0)	0 (0.0)	0 (0.0)	2 (0.4)	0 (0.0)	2 (0.4)	2 (0.1)

**Table 2:** Overall knowledge of TB by selected characteristics.

Characteristics	High TB knowledge	Low TB knowledge	*p* value
Sex			<0.001
Males	816 (64.2)	455 (35.8)	
Females	793 (57.8)	578 (42.2)	
Marital status			<0.0001
Single	441 (73.4)	160 (26.6)	
Married	1,106 (57.5)	818 (42.5)	
Divorced/separated/other	57 (52.3)	52 (47.7)	
Age categories			<0.003
16–29	654 (63.7)	372 (36.3)	
30–44	624 (60.5)	408 (39.5)	
45–59	267 (58.8)	187 (41.2)	
60 years and above	53 (46.9)	60 (53.1)	
Study sites			<0.0001
Akwa Ibom	371 (72.5)	141 (27.5)	
Benue	319 (64.1)	179 (35.9)	
Ebonyi	185 (45.7)	220 (54.3)	
Gombe	235 (53.2)	207 (46.8)	
Katsina	203 (53.6)	176 (46.4)	
Ondo	296 (72.7)	111 (27.3)	
Educational status			<0.0001
No formal education/Quranic	210 (37.6)	348 (62.4)	
Primary	287 (54.3)	242 (45.7)	
Secondary	671 (66.8)	334 (33.2)	
Tertiary	434 (81.0)	102 (19.0)	
Occupation			<0.0001
Not employed	154 (53.8)	132 (46.2)	
Student	243 (71.9)	95 (28.1)	
Self-employed/business owner	836 (55.1)	681 (44.9)	
Formal employment	309 (79.0)	82 (21.0)	

**Table 3:** Communities attitudes towards TB by selected characteristics.

Characteristics	Good treatment seeking behavior for TB	Poor treatment seeking behavior for TB	Pearson *χ*^2^ *p* value
Sex			<0.237
Males	915 (72.0)	356 (28.0)	
Females	1,015 (74.0)	356 (26.0)	
Marital status			<0.025
Single	450 (74.9)	151 (25.1)	
Married	1,383 (71.9)	541 (28.1)	
Divorced/separated/other	90 (82.6)	19 (17.4)	
Age categories			<0.102
16–29	728 (71.0)	298 (29.0)	
30–44	756 (73.3)	276 (26.7)	
45–59	348 (76.7)	106 (23.3)	
60 years and above	87 (77.0)	26 (23.0)	
Study sites			<0.0001
Akwa Ibom	428 (83.6)	84 (16.4)	
Benue	456 (91.6)	42 (8.4)	
Ebonyi	326 (80.5)	79 (19.5)	
Gombe	187 (42.3)	255 (57.7)	
Katsina	353 (93.1)	26 (6.9)	
Ondo	180 (44.2)	227 (55.8)	
Educational status			<0.0001
No formal education/Quranic	366 (65.6)	192 (34.4)	
Primary	389 (73.5)	140 (26.5)	
Secondary	722 (71.8)	283 (28.2)	
Tertiary	444 (82.8)	92 (17.2)	
Occupation			<0.002
Not employed	199 (69.6)	87 (30.4)	
Student	245 (72.5)	93 (27.5)	
Self-employed/business owner	1,086 (71.6)	431 (28.4)	
Formal employment	315 (80.6)	76 (19.4)	
Knowledge of TB			<0.0001
Low level of knowledge	641 (62.0)	393 (38.0)	
High level of knowledge	1,289 (80.1)	320 (19.9)	

**Table 4:** Crude and adjusted ratios of overall knowledge of TB.

Characteristics	Crude odds ratios	*p* value for crude odds ratios	Adjusted odds ratios	*p* value for adjusted odds ratios
Sex				
Males (ref.)	*1*		*1*	
Females	1.136 (0.964–1.340)	NS	1.113 (0.914–1.354)	NS
Marital status				
Married (ref.)	*1*		*1*	
Other	1.190 (0.985–1.438)	NS	1.019 (0.808–1.284)	NS
Age categories				
16–29 (ref.)	*1*		*1*	
30–44	1.080 (0.912–1.279)	NS	1.019 (0.808–1.284)	NS
45–59	0.900 (0.725–1.116)	NS	1.042 (0.780–1.392)	NS
60 years and above	0.726 (0.492–1.071)	NS	0.785 (0.491–1.257)	NS
Educational status				
No formal education/Quranic (ref.)	*1*		*1*	
Primary	0.921 (0.751–1.129)	NS	1.404 (1.062–1.857)	*∗*
Secondary	1.428 (1.200–1.699)	*∗∗∗*	2.165 (1.651–2.839)	*∗∗∗*
Tertiary	1.710 (1.370–2.135)	NS	2.833 (2.006–4.002)	*∗∗∗*
Occupation				
Not employed (ref.)	*1*		*1*	
Student	0.944 (0.738–1.206)	NS	0.746 (0.490–1.137)	NS
Self-employed/business owner	0.868 (0.731–1.032)	NS	0.892 (0.646–1.230)	NS
Formal employment	1.494 (1.666–1.915)	*∗∗*	0.906 (0.595–1.381)	NS
Knowledge of TB				
Low level of knowledge (ref.)	*1*		*1*	
High level of knowledge	0.474 (0.401–0.561)	NS	0.585 (0.481–0.711)	NS

NS: not significant. ^*∗*^Minimal significance; ^*∗∗*^higher level of significance; ^*∗∗∗*^very significant.

## Additional Points

*Limitations*. The study has certain limitations. The data used was obtained through a cross-sectional survey, so causal relationship cannot be ascertained. Furthermore, information on knowledge was based on self-reported responses by the respondents. In order to assess the continuous level of TB knowledge among the general population, a follow-up study at more time points would be desirable. The collection of data that could be used to estimate wealth status of the respondents would also be required, to help identify TB knowledge gaps among the different economic groups in future follow-up studies.
